# Radiofrequency Ablation of Benign Thyroid Nodules: Preliminary Outcomes of an Endocrine Surgery Unit

**DOI:** 10.7759/cureus.96863

**Published:** 2025-11-14

**Authors:** Miguel A Almeida, Tiago Pimenta, Pedro S Couto, Silvestre Carneiro

**Affiliations:** 1 General Surgery, São João Local Health Unit, Porto, PRT; 2 Surgery, Faculty of Medicine, University of Porto, Porto, PRT

**Keywords:** ablation, moving-shot, radiofrequency, rfa, thyroid nodule

## Abstract

Introduction

Radiofrequency ablation (RFA) is a minimally invasive alternative for the management of benign thyroid nodules in patients with symptomatic or enlarging lesions who seek to avoid the risks and morbidity associated with surgery. Our aim was to evaluate the initial RFA results of our endocrine surgery unit.

Methods

We analyzed our early RFA outcomes from November 2022 to November 2024. The VIVA RF system (STARmed Co. Ltd., Goyang-si, GG, KOR) was used, with an internally cooled 18-gauge electrode and a 10-mm active tip. The ultrasound (US)-guided trans-isthmic approach and the moving-shot technique were used in all patients after two confirmed Bethesda II cytologies.

Results

We identified 33 cases, mostly women (93.9%), with a mean age of 54 years old. Surgical indications were restricted to compressive symptoms (19/33, 57.6%), cosmesis (4/33, 12.1%), or both (10/33, 30.3%). The nodules were classified per the European Thyroid Imaging and Reporting Data System (EU-TIRADS): category 2 (2/33, 6.1%), category 3 (25/33, 75.8%), and category 4 (6/33, 19.2%). The average nodule volume was 12 mL. The mean energy delivered and duration were 16150 J (± 10836) and 7.2 min (± 4.3), respectively. We report three cases (9.0%) of postoperative morbidity: a subcutaneous hematoma, a Horner syndrome, and a patient with transient hoarseness. At three months, the mean volume reduction ratio (VRR) was 40.4%, while the six-month VRR was 47.1%.

Conclusion

Our initial experience demonstrated a near 50% volume reduction at six months. The RFA’s low complication rate, coupled with the freedom to perform the procedure with local anesthesia, makes it an attractive option. Regrowth and the need for reintervention are possible. Further follow-up is necessary to assess long-term efficacy and recurrence.

## Introduction

Thyroid nodules are a very frequent finding in adults, being more common in women and older-aged patients, with a variable estimated prevalence depending on the detection method used in epidemiological studies [1,2]. Palpable nodules are present on examination in approximately 4% to 7% of the general population. Recently, clinically inapparent nodules have been detected with increasing frequency by ultrasound (US) and other sensitive imaging techniques in up to two-thirds of randomly selected individuals [3,4].

Despite a minority of patients who harbor malignancy, most nodules are cytologically benign, asymptomatic, tend to be incidentally found during imaging for unrelated reasons, and can be safely observed long term [5]. Even so, benign thyroid nodular disease accounting for compressive neck symptoms and/or cosmetic deformity should be considered for treatment [6,7].

The safety and efficacy of thyroid surgery are well-documented, particularly in the hands of high-volume experienced surgeons, and it has traditionally been the main treatment choice [8,9]. Nevertheless, many patients with benign disease delay or avoid resection due to the operation’s risk of complications (life-threatening neck hematoma, recurrent laryngeal nerve injury, and hypoparathyroidism) and the potential requirement for lifelong hormone supplementation [9,10]. To overcome these significant concerns, thermal ablative (TA) techniques have emerged as minimally invasive outpatient procedures. These gather the advantages of not requiring general anesthesia, leaving minimal scarring, allowing for faster recovery, and preserving euthyroidism [11].

Thermal ablation occurs as a consequence of high temperatures, which lead to focal cellular apoptosis, coagulative necrosis, and subsequent irreversible nodule damage and shrinkage [6,9,10]. Regarding radiofrequency ablation (RFA), a probe percutaneously placed within the target lesion produces a high-frequency alternating current (200 to 1200 kHz) that generates frictional agitation at the ionic level and conductive heat by the Joule effect, reaching temperatures up to 100ºC [12,13]. The goal is a selective and targeted nodule ablation sparing critical adjacent structures (i.e., the inferior laryngeal nerve, trachea, or carotid artery) [14]. This single-center study aimed to evaluate the initial RFA results of our endocrine surgery unit. We focused on assessing the efficacy, safety, and feasibility of RFA in reducing the volume of benign thyroid nodules as a minimally invasive alternative to conventional thyroid surgery.

This article was previously presented as an oral communication at the 11th Conference of the European Society of Endocrine Surgeons (ESES) in Izmir, Turkey, on May 23rd, 2025.

## Materials and methods

We conducted a retrospective cohort study including all consecutive cases of benign thyroid nodules subjected to RFA at our tertiary care center since we began to use this technique. We analyzed data from November 2022 to September 2024, identified through electronic medical records. A review of demographic, clinical, sonographic, and surgical data was performed.

Ultrasound assessment of thyroid nodules and stratification of requirements for fine-needle aspiration biopsy (FNAB) were per the European Thyroid Imaging and Reporting Data System (EU-TIRADS). Nodule composition was classified as 'mixed' when a cystic component constituted >10% of the nodule volume [15]. Patients were selected for treatment if they presented cervical compression complaints, poor cosmesis, or both, after two FNABs with proven benignity per the Bethesda II cytology. Additionally, normal calcitonin levels were confirmed to exclude the remote possibility of medullary thyroid carcinoma among low- or intermediate-risk nodules on sonography.

We estimated the baseline nodule’s volume using the ellipsoid formula (i.e., D1 × D2 × D3 × 0.52) [8]. As per Deandrea et al., we calculated the minimal required energy to obtain a nodular volume reduction above 50% by determining 1500 J × nodule volume mL [16]. All procedures were performed by multiple trained surgeons of our unit. The VIVA RF system (STARmed Co. Ltd., Goyang-si, GG, KOR) was used. It contains an internally cooled 18-gauge electrode and a 10 mm active tip. A US-guided trans-isthmic approach and the moving-shot technique were used in all patients under local anesthesia with 2% lidocaine [17]. Volume reduction ratio (VRR) at three months and six months was calculated with the formula: initial volume (mL) − final volume (mL) x 100 / initial volume (mL). Written informed consent was obtained from the patients to use their clinical data for research purposes. Statistical analysis was executed with SPSS Statistics version 29 (IBM Corp., Armonk, NY, USA).

## Results

A total of 33 cases were identified and included during a 22-month study period. Among the cohort, the vast majority of cases concerned women (93.9%) with a mean age of 54 years old (range 20 to 81). Surgical indications were restricted to compressive symptoms (19/33, 57.6%), cosmesis (4/33, 12.1%), or both (10/33, 30.3%). Regarding nodule location, the left lobe was the predominant site (20/33, 60.6%) in comparison to the right lobe (12/33, 36.4%), besides one single isthmic nodule (1/33, 3.0%). On the topic of US features, nodules were classified as EU-TIRADS 2 (2/33, 6.1%), 3 (25/33, 75.8%), and 4 (6/33, 19.2%), with balanced numbers in terms of structural composition, i.e., solid (15/33, 45.5%) or mixed (18/33, 54.5%). The average baseline nodule volume was 12.1 mL. The mean energy delivered and radiofrequency firing duration were 16150 J (± 10836) and 7.2 minutes (± 4.3), respectively. We calculated our energy delivered per nodule volume to be 1334.7 J/mL.

**Figure 1:**
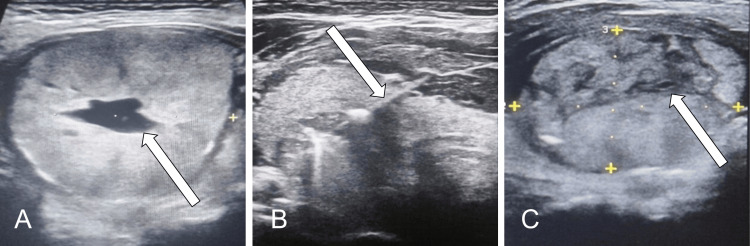
Preprocedural, intraprocedural and postprocedural ultrasound A: A right-sided thyroid nodule with mixed composition and a central cystic component (arrow) measuring 37 x 35 x 26 mm (EU-TIRADS 3); B: Moving-shot technique where an in-plane oblique trans-isthmic approach is used to guide the electrode (arrow); C: Immediate appearance of the nodule after treatment with residual hypoechogenic areas (arrow) EU-TIRADS: European Thyroid Imaging and Reporting Data System

We report three cases (9.0%) of postoperative morbidity: a subcutaneous hematoma, a Horner syndrome, and a patient with transient hoarseness. The subcutaneous hematoma was minor and required only observation, a cold compress, and analgesia. The Horner syndrome showed significant improvement after four months of targeted facial rehabilitation. The transient hoarseness had resolved by the first postoperative consultation at the one-month mark. We registered a mean follow-up of 8.8 months. At the three-month US evaluation, the mean VRR was 40.4%, while the six-month VRR was 47.1%. Throughout the study period, only one patient required a second ablation due to nodule regrowth (3.0%). A detailed overview of demographics, nodule characteristics, sonography, and the obtained interventional outcomes is exhibited in Table 1. Thyroid US examples of the preprocedural, intraprocedural, and postprocedural phases are displayed in Figure 1. 

**Table 1:** Demographics, nodule characteristics, sonography, interventional data and outcomes (total n=33) EU-TIRADS: European Thyroid Imaging and Reporting Data System, VRR: Volume reduction ratio

Parameters		Number	Mean/percentage
Age (years), mean (range)		54	20-81
Gender (n, %)	Female	31	93.9%
	Male	2	6.1%
Surgical indication (n, %)	Compressive symptoms	19	57.6%
	Cosmesis	4	12.1%
	Both	10	30.3%
Nodule location (n, %)	Right lobe	12	36.4%
	Left lobe	20	60.6%
	Isthmus	1	3.0%
Composition (n, %)	Solid	15	45.5%
	Mixed	18	54.5%
EU-TIRADS (n, %)	2	2	6.1%
	3	25	75.8%
	4	6	18.2%
Baseline nodule volume (mL), median (IQR)		12.1	7.5-19.8
Energy delivered (joules), mean (σ)		16150	10836
Firing duration (min), mean (σ)		7.2	4.3
Postoperative morbidity (n, %)		3	9.0%
Subcutaneous hematoma		1	3.0%
Transient hoarseness		1	3.0%
Horner syndrome		1	3.0%
VRR (%), median (IQR)	Three-month VRR	40.4	28.5-55.0
	Six-month VRR	47.1	33.2-62.5
Follow-up (months), mean (σ)		8.8	5.8

## Discussion

Radiofrequency ablation has been progressively used and refined since the mid-2000s and is currently established as the dominant TA approach, upon consistent efficacy and durability [10]. Among different modalities, laser thermal ablation (LTA) is well-tolerated but comparatively provides slightly lower VRR, while microwave ablation (MWA) can be beneficial for bigger nodules due to higher and uniform intranodular temperatures, resulting in a more predictable ablation zone [18,19]. In contrast, the cost and limited access to high-intensity focused ultrasound (HIFU) remain barriers to its widespread adoption [20].

A systematic review and meta-analysis on RFA by Trimboli et al. disclosed an overall VRR at six, 12, and 24 months of 68%, 75%, and 87%, respectively [21]. We consider our six-month VRR of 47.1% both a successful short-term outcome and an encouraging preview of the expected final volume reduction, as it typically continues for up to two years. Still, reluctance to deliver more energy during the early stage of the learning curve may have vaguely compromised preliminary results, given that the success of RFA is positively correlated to energy delivered per nodule [22]. Our energy delivery per mL of nodule volume was ~1335 J/mL (16150 J/12.1 mL), which is slightly below the threshold proposed by Deandrea et al., supporting the notion of a thoughtful approach [16].

Concerning the thyroid tissue architecture, we noticed a near-equal distribution of solid (45.5%) and mixed (54.5%) nodules. Unlike nodules with a predominantly cystic component, which tend to respond most dramatically to RFA, solid tissue can be more resistant to ablation and require higher energy for optimal necrosis [20,23]. Therefore, the structural composition of treated nodules might have also affected our initial VRR.

Nodule regrowth and the need for retreatment are possible, occurring more frequently in nodules closely located to vital structures or large nodules wherein effective treatment of all viable tissue can be challenging [10,24]. The available studies on considerable series with long-term follow-up (at least two years) reveal regrowth percentages ranging from 4.6% to 34%, with at least one re-intervention required in up to 23.8% of patients [25]. In our cohort, only one patient (3.0%) needed two sessions due to volume regain, timed one year apart, which is a favorable finding. However, it is likely that our reassessment timings were too fleeting to detect regrowth, thus underestimating true incidence.

Intraoperatively, we adopted the moving-shot technique proposed by Jeong et al. instead of the fixed-needle technique generally used for liver tumors [17]. Its purpose is to avoid the dangerous, prolonged fixation of the electrode near noble surrounding structures. It presupposes compartmentalizing the thyroid nodule into multiple small virtual units and performing sequential ablation of each unit on the move [13]. The buildup of microbubbles increases resistance and creates a bright ultrasound signal. The gas created then eventually disperses, leaving the treated area with a residual hypoechogenic appearance [11].

Regarding the safety profile of TA approaches, all existing modalities are recognized as low-risk with no significant differences between RFA, LTA, MWA, and HIFU. Complications are significantly lower than with surgery, most adverse events being minor and temporary, whilst the prospect of hypothyroidism, permanent nerve injury, or significant hemorrhage is minimal [14,20]. With respect to RFA, the reported rate of minor complications is roughly 4%, and major complications 1.1% to 1.4% [11].

Besides registering no permanent morbidity, our transient morbidity rate of 9% is comparable to similar studies. We observed one case of self-limited subcutaneous hematoma, a known risk of RFA, generally secondary to bleeding after inadvertently transversing an anterior jugular or a pericapsular thyroid vein [10,26]. Additionally, we detected a patient with transient hoarseness, probably owing to unintentional heat transmission to the recurrent laryngeal nerve (RLN), that fully resolved conservatively. Voice changes may also be caused by RLN compression from intranodular hemorrhage, nonetheless [27]. Furthermore, we account for a case of Horner syndrome, which is a rare complication with an estimated incidence of 0.1% to 1.5%. It results from thermal injury to the cervical sympathetic chain, most often when the ablation zone is close to the middle cervical ganglion or the inferior thyroid artery. Its clinical significance includes the classic triad of ipsilateral ptosis, miosis, and facial anhidrosis [20,28]. Once objective findings are frequently mild, Horner syndrome is prone to being underreported. Heightened vigilance and future large-scale studies are crucial to ascertain more precise epidemiological data.

From an operational standpoint, our experience confirms the feasibility of integrating RFA into a busy endocrine surgery unit. The procedures were successfully performed in an outpatient setting under local anesthesia, with an average active treatment time of just over seven minutes. This competence minimizes resource utilization compared to traditional surgery, which requires more operating room time, general anesthesia, and often, an inpatient stay.

This study has a few inherent limitations that must be mentioned. To begin with, it was developed with a retrospective design. The sample size, while informative for a first experience, is relatively small. The follow-up period is short for a definitive assessment of long-term efficacy, particularly for detecting nodule regrowth, which most commonly occurs two to three years post-procedure.

Although beyond the scope of our investigation, it should be noted that RFA is gaining acceptance in the management of papillary thyroid cancer (PTC) [9,11,20]. In fact, its role remains an area of active debate and a matter of significant divergence between Eastern and Western practices. While the Asians (most notably China and South Korea) have adopted broader indications, North America and Europe remain cautious, prioritizing proven long-term oncologic control. A recently issued international expert consensus recommends thermal ablation as a first-line treatment for T1aN0M0 PTC and as an alternative treatment for T1bN0M0 PTC if patients are uncomfortable with active surveillance or unfit for surgery [29]. Conversely, the 2025 American Thyroid Association (ATA) guidelines on differentiated thyroid cancer are narrower, limiting percutaneous ablation for selected patients with T1aN0M0 PTC to second-line exclusively [30]. Moreover, RFA can be helpful in palliation by providing symptomatic relief and mitigating local disease progression when a complete cure is not realistic [26].

## Conclusions

Our initial experience establishes RFA as a safe, effective, and feasible minimally invasive therapy for benign thyroid nodules in an endocrine surgery unit. It successfully alleviates compressive symptoms and cosmetic concerns while obviating the risks of surgery and general anesthesia. The transient complication rate was low and acceptable. As we progress beyond the learning curve, we anticipate our VRR will approach those reported in the literature. Future efforts will focus on optimizing energy delivery, refining patient selection criteria, and prospectively evaluating long-term outcomes and patient satisfaction to fully establish the role of RFA within our treatment arsenal.
